# Editorial: Alternative Therapeutics against MDR Bacteria – “Fighting the Epidemic of Antibiotic Resistance”

**DOI:** 10.3389/fmicb.2016.01559

**Published:** 2016-10-07

**Authors:** Marta Martins, Matthew P. McCusker

**Affiliations:** ^1^Department of Microbiology, Moyne Institute of Preventive Medicine, School of Genetics and Microbiology, Trinity College Dublin, The University of DublinDublin, Ireland; ^2^School of Food Science and Environmental Health, College of Sciences and Health, Dublin Institute of TechnologyDublin, Ireland

**Keywords:** multidrug-resistance, drug discovery, alternative therapy, novel compounds, rational design, nanotechnology, drug repurposing

The introduction of the first antibiotic, followed by the “golden age” of antibiotic discovery was expected to herald the end of infectious diseases. However, microbial evolution in response to antibiotic selection pressure has ended that expectation. The rise of resistance coincided with a decline in the discovery of new antibiotics. With some infections now essentially untreatable, initiatives are being put in place to redress this situation.

Among the *Enterobacter* species, resistance in *Enterobacter aerogenes* and *E. cloacae* is rising. Davin-Regli and Pagès provide an overview of the factors that contribute to this and to the dissemination of *Enterobacter* spp.. These authors discuss how the prevalence of multiresistant isolates of *Escherichia coli* and *Klebsiella pneumoniae* from major international clones has increased during the last decade while the incidence of *E. aerogenes*, which has proven unable to develop a major clone to date, has declined significantly. This is supported by recent findings that diverse fitness associated with resistance to fluoroquinolones influenced the selection of these major clones (Fuzi, 2016). Additionally, these clones retain fitness by developing high-level fluoroquinolone resistance without using energy-consuming efflux (Tóth et al., 2014; Johnson et al., 2015). The fact that active efflux systems are widespread in *E. aerogenes* supports this concept (Chevalier et al., 2008).

In the battle against these and other pathogens, rapid detection and characterization of their drug resistance profile is crucial. Nanotechnology-based approaches have allowed for the development of fast and sensitive detection methods for various pathogens. In their review, Baptista and co-workers discuss the use of gold nanoparticles to screen molecular signatures of drug resistance (Veigas et al.). These authors provide a critical evaluation of current and future developments of technologies assisting pathogen identification and characterization of antibiotic resistance profiles.

The use of antimicrobial peptides has been investigated due to their broad-spectrum activity. Roy and co-workers reported the use of a modified broth of *Bacillus subtilis* showing activity against multidrug-resistant *Staphylococcus aureus, S. epidermidis, Streptococcus pyogenes* and *Enterococcus faecalis* (Chalasani et al.). This and other lead molecules are needed to treat different types of infections caused by pathogens, like *S. aureus*. In prosthetic vascular grafts *S. aureus* leads to high morbidity and mortality. Due to its role in virulence and biofilm formation, the Staphylococcal accessory regulator A (SarA) constitutes an attractive target for anti-biofilm agent development. Princy and co-workers performed a structure-based screening of lead compounds to identify novel small inhibitors targeting *SarA* (Arya et al.). A top-hit selective inhibitor was reported to prevent attachment of *S. aureus* to an epithelial cell line and inhibit the colonization of multidrug-resistant *S. aureus* in an animal model. In addition, they performed an *in silico* design of a hybrid molecule composed of a molecule screened from *M. dubia* and a modified SarA-based inhibitor (Balamurugan et al.). They aimed to develop a molecule targeting SarA. Based on their results, the hybrid molecule alone and in combination with gentamicin was able to reduce the biofilm structure and kill the bacteria. This can be a promising candidate molecule to be used alone or, in combination with an antibiotic to treat urinary tract infections caused by *S. aureus*.

Combination therapy has been explored for the treatment of infections caused by *Mycobacterium tuberculosis*. *M. tuberculosis* intrinsic resistance to antimicrobials has been attributed mainly to reduced permeability of the cell wall. Allied to this, efflux systems extrude drugs from the bacteria. As these systems play a crucial role in mediating multidrug-resistance, inhibiting efflux is an attractive approach. Viveiros and co-workers performed an elegant study in clinical isolates of multidrug-resistant *M. tuberculosis* using single combinations of antituberculosis drugs and efflux inhibitors (Coelho et al.). In this study, synergies between isoniazid, rifampicin, amikacin and ofloxacin, and the inhibitors verapamil, thioridazine and chlorpromazine were reported. Taken together, their results confirm that in multidrug-resistant *M. tuberculosis* the intrinsic efflux activity contributes to the overall resistance and the inhibition of efflux can enhance the effect of antibiotics.

The empty drug discovery pipeline has lead to a re-focus of research into finding new uses for existing drugs. Anticancer, antifungal, anthelmintic and anti-inflammatory drugs with antimicrobial properties have been discovered. García-Contreras and co-workers discuss alternative approaches to treat bacterial infections by repurposing existing drugs as antibiotics or virulence inhibitors (Rangel-Vega et al.). The use of anticancer gallium compounds or drugs such as niclosamide are some examples. Other drugs such as 5-fluorouracil, azithromycin, ceftazidime and ciprofloxacin, are known to decrease the expression of *quorum sensing* controlled virulence factors.

In this battle against resistance the use of bioengineered bacteriophage lysins is being explored. These highly specific hydrolases present broader killing spectrum. When engineered, they can be used as recombinant enzymes against Gram-positive bacteria, causing rapid lysis upon direct contact with peptidoglycan. This ability has laid the foundation for exploiting them as powerful antimicrobials. Additionally, they can selectively target specific pathogens without affecting surrounding microbiota. In their mini-review, Wei and co-workers summarize the current knowledge on lysins and their possible uses as antimicrobials (Yang et al.). They review their modular structure, mode of action and modifications to improve their lytic activity.

Siderophores are ferric ion specific chelating agents, secreted by bacteria and fungi under low iron conditions. Their main role is to scavenge the essential mineral from the surrounding environment, making it available to the microbial cell. Due to their essential role in virulence and microbial survival, siderophores have become subject of interest for use as target delivery of antimicrobials. In their general commentary, de Carvalho and Fernandes discuss the “Trojan Horse” approach, i.e., the use of siderophores to tackle multidrug-resistance. They present an overview of approaches that take advantage of the iron transport system and of the work being conducted to improve antibiotic uptake by pathogenic bacteria, by designing siderophore-antibiotic conjugates.

In summary, this research topic focused on new methodologies to identify antimicrobial resistance genes, discovery of new molecules and repurposing of old ones. In addition, novel therapies combining “old” antibiotics and efflux inhibitors could be an avenue to explore in the future treatment of multidrug-resistant infections.

## Author contributions

MM and MPM contributed equally to the writing and editing of this editorial.

## Funding

Work in the lab of MM on reversal of multidrug-resistance and novel compounds is funded by the Irish Research Council under the employment based postgraduate programme EBPPG/2015/233.

### Conflict of interest statement

The authors declare that the research was conducted in the absence of any commercial or financial relationships that could be construed as a potential conflict of interest.
